# A computational model of the integration of landmarks and motion in the insect central complex

**DOI:** 10.1371/journal.pone.0172325

**Published:** 2017-02-27

**Authors:** Alex J. Cope, Chelsea Sabo, Eleni Vasilaki, Andrew B. Barron, James A. R. Marshall

**Affiliations:** 1 Department of Computer Science, University of Sheffield, Sheffield, South Yorkshire, United Kingdom; 2 Sheffield Robotics, Sheffield, South Yorkshire, United Kingdom; 3 Macquarie University, Sydney, Australia; University of Sussex, UNITED KINGDOM

## Abstract

The insect central complex (CX) is an enigmatic structure whose computational function has evaded inquiry, but has been implicated in a wide range of behaviours. Recent experimental evidence from the fruit fly (*Drosophila melanogaster*) and the cockroach (*Blaberus discoidalis*) has demonstrated the existence of neural activity corresponding to the animal’s orientation within a virtual arena (a neural ‘compass’), and this provides an insight into one component of the CX structure. There are two key features of the compass activity: an offset between the angle represented by the compass and the true angular position of visual features in the arena, and the remapping of the 270° visual arena onto an entire circle of neurons in the compass. Here we present a computational model which can reproduce this experimental evidence in detail, and predicts the computational mechanisms that underlie the data. We predict that both the offset and remapping of the fly’s orientation onto the neural compass can be explained by plasticity in the synaptic weights between segments of the visual field and the neurons representing orientation. Furthermore, we predict that this learning is reliant on the existence of neural pathways that detect rotational motion across the whole visual field and uses this rotation signal to drive the rotation of activity in a neural ring attractor. Our model also reproduces the ‘transitioning’ between visual landmarks seen when rotationally symmetric landmarks are presented. This model can provide the basis for further investigation into the role of the central complex, which promises to be a key structure for understanding insect behaviour, as well as suggesting approaches towards creating fully autonomous robotic agents.

## Introduction

The central complex (CX) lies at the centre of the insect brain as well as that of other arthropods. It is highly conserved across insect species [1, 2] and is the target of sensory convergence [3]. Furthermore, it has been implicated in many insect behaviours including locomotion [4, 5], courtship [2], visual pattern memory [6], visual place learning [7], and polarisation detection for the celestial compass [8, 9]. It is therefore surprising that little is known about the computational function of this structure. Recent work has begun to open the door on this enigmatic region of the the insect brain, and here we aim to structure what is known about the function of one set of neurons in CX into a well constrained computational model. This model can then provide a foundation for exploration of the entire CX. As well as understanding brain function, knowledge of the role of this structure could have importance in the development of truly autonomous robotic agents exhibiting the type of visual place learning attributed to the CX [7].

Neurons that represent an organism’s orientation within the world (a ‘neural compass’) are well established in vertebrates, such as the rat [10]. Recent work has shown that similar cells can also be found in the CX of the considerably simpler fruit fly, *Drosophila melanogaster* [11], and the cockroach *Blaberus discoidalis* [12]. Such cells are likely to exist across many insect and arthropod species given the high degree of conservation of the central complex neuropils. These cells could allow an insect to represent their current heading, and form the basis of a variety of complex behaviours including learned orientations and navigation. The mechanisms behind the activity of these heading cells are currently unknown, and understanding them would provide insight into the role of the central complex in guiding behaviour [13], as well as insights into how neural compasses are involved in generating behaviour in vertebrates. Here we will focus on the evidence from *Drosophila* rather than the cockroach, as it is arguably the more detailed evidence base.

The recent findings in *Drosophila* were made in restrained flies walking on a rotating air-supported ball in a virtual arena. Seelig and Jayaraman [11] demonstrated that the activity of one class of neurons (EBw.s, using the notation from [11])) in the toroidal ellipsoid body (EB) of the CX formed a bump, which tracked the location of a landmark (a vertical bar) over the course of over two minutes. Furthermore this bump moved to track the rotational motion of the insect when in complete darkness, albeit with growing inaccuracy. These experiments therefore demonstrate that the location of the bump can be driven by accurate, likely landmark-based, positional processes or less accurate motion-based processes, with the positional process dominating.

The experiments of Seelig and Jayaraman also indicate that the mapping of the landmarks’ receptive fields (RFs) onto the EBw.s. neurons are plastic rather than fixed. Firstly, in the animals tested experimentally there was an offset between the orientation of the landmark relative to the heading of the fly, and the orientation of the neural compass relative to the heading of the fly. This offset persists for long periods of time (i.e. minutes (personal communication, Turner-Evans, March 21st 2016)), but can shift between experiments, and each fly has a different offset. This offset implies that the landmarks are not bound to the EB ring by static connections. More experimental evidence for learned landmark mappings arises from the nature of the stimuli Seelig and Jayaraman presented to their flies. The stimulus LED array covered 270° of the visual field of *Drosophila*, yet when a stimulus rotated to the rear of the array it transitioned the 90° gap instantaneously. This gave the *Drosophila* a visual world that wrapped to 270° but which was then mapped onto the full 360° of the EB ring. Combined these results show that landmarks can be remapped both in offset and spacing onto the EB ring, however the mechanisms and pathways underlying this plasticity are completely unknown.

There is also neuroanatomical evidence that supports the plasticity of the landmark RF. The most likely landmark input cells are the R4 neurons which project from the lateral triangular regions (LTR, lateral accessory lobe) to the EB, and have been shown to have receptive fields that cover specific regions of the visual field [14]. The arborisations of the R4 neurons permeate large regions of the EB ring, rather than specific regions corresponding to the positions of their RFs in the fly’s visual field. Interestingly, most R-type neurons stain strongly for GABA, implying an inhibitory effect, with fewer staining for excitatory neurotransmitters. We will show that this distribution of neurotransmitters can serve an important computational role.

These experiments with walking *Drosophila* follow from a lineage of behavioural experiments using restrained flying *Drosophila* [15–18]. Of these the most relevant involves training flies to respond to stimuli (green and blue global illumination) by fixed angular rotations [17]. The flies are trained in an environment consisting of a regular grating and therefore containing no positional landmarks. Notably, recent data have shown that such behaviours also require a functioning CX to operate [18]. These experiments require that the fly is able to integrate its angular motion using visual information, and can use this information to guide orienting behaviour.

This behavioural evidence for angular motion integration in the CX requires a suitable wide-field motion signal, which must be monotonically proportional to the speed of movement (or an efference copy or proprioceptive signal calibrated by a visual motion signal). For this we have two candidates: the optomotor response and the angular velocity response. It has been well established in most insects that there exists a motion detection response formed by simple correlation detectors and tuned to the temporal frequency of contrast edges passing the detector. This is termed the optomotor response [15, 19]. This pathway is unsuitable for driving angular motion integration as the response depends upon the spatial frequency of the environment, and thus will be inconsistent in the magnitude of response to a given rotation. The second response is less well established however significant evidence exists in both bees [20–22] and behaviourally in *Drosophila* [23] for its existence. This response reports the angular velocity (AV) of motion independent of spatial frequency and contrast, and as such is suitable for driving angular motion integration. Our recent model proposes a neural circuit for this response [24] which we will use in the subsequent modelling work. We assume a pathway from the optic neuropils to the CX for this information based on the behavioural evidence and the existence of anatomical evidence for a visual pathway to CX via the lateral accessory lobe (LAL) [25].

We are therefore interested in how these two bodies of evidence, angular motion integration from vision and a neural compass in the CX, converge. If the learned angular rotations are performed using the CX ellipsoid body bump activity and can be driven by angular motion integration then how do positional cues and motion information interact? What are the computational advantages of having two systems for tracking orientation visually? Finally, how can landmarks be associated with compass positions on the EB ring?

## Model and methods

### Modelling tools

The model was created and simulated using the SpineML toolchain [26] and the SpineCreator graphical user interface [27]. These tools are open source and installation and usage information can be found on the SpineML website at http://spineml.github.io/. Visual input is provided using our raytracer ‘Beeworld’, described in more detail in the Supporting Information of Cope et al [24].

### Model variants

To investigate the behaviour of the model we present two variants. One variant is used in the initial experiments and we set the positional input to the neural compass to have fixed weights and retinotopy. This allows us to investigate the idealised behaviour of the compass with positional and motion system input as well as the behaviour with motion or positional system input independently. This variant is described as ‘fixed weight’. The other is the full version of the model where we introduce plasticity to the weights governing the positional input to the neural compass. This allows the model to learn weights that remap the visual field onto the neural compass and can therefore reproduce the experimental findings of Seelig and Jayaraman [11]. This variant is described as ‘plastic weight’.

### Motion detection

**Fig 1:**
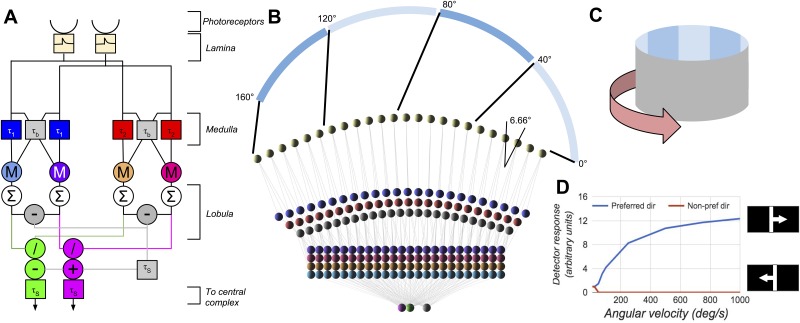
The motion detection section of the model for one eye. **A** shows the structure of a detector unit spanning two neighbouring ommatidia, with purple and green outputs responding to angular velocity (AV) in the progressive and regressive directions respectively. *M* denotes multiplication, */* denotes division, the *∑* denotes summation over the entire eye. The *τ* are the time constants of leaky integrators. **B** shows the horizontal layout of the units and connections for a single eye, and the mapping from visual space to the model photoreceptors, with 0° representing directly in front of the modelled fly. **C** shows a diagram of the visual environment, which can be rotated. **D** shows the response of the angular velocity detector to a single 11.5° bar moving across the field of view at different angular velocities. The parameters of the model are (full details can be found in [24]): *τ*_1_ = 5*ms*, *τ*_2_ = 15*ms*, *τ*_*b*_ = 1*ms*, *τ*_*S*_ = 10*ms*, and control the range and nature of the angular velocity response of the AVDU.

The motion detection circuit used in this model comprises our earlier model [24] adapted with an ommatidial pattern with similar ommatidia numbers and density to that found in wild-type *Drosophila* [28]. This pattern consists of 24 horizontal by 32 vertical ommatidia per eye in a grid. This ommatidial grid covers different fields of view for the initial experiments where the behaviour of the model without plasticity is tested (fixed weight model, Experiments 1 & 2) and the experiments where plasticity between the visual RF and the CX is present (plastic weight model, Experiment 3). For the fixed weight experiments the field of view is 360° horizontally by 180° vertically. The full 360° field of view is used in the fixed weight experiments so that the accuracy is not affected by the 90° blind spot. For the plastic weight experiments the field of view is 270° horizontally by 180° vertically. For the plastic weight experiments, in keeping with the experimental paradigm of Seelig and Jayaraman [11], the stimuli transit the 90° blind spot instantaneously. For each adjacent pair of ommatidial locations in the horizontal direction there are correlation detectors with two time constants, and in addition there are two of these pairs of detectors per pair of ommatidial locations, one preferring progressive motion (i.e. forward flight) and one preferring regressive motion (i.e. backward flight). This gives four correlation detectors per ommatidial pair in total. The outputs of these correlation detectors are summed across each of the left and right eyes and combined as in Fig 1 to produce angular velocity detectors (AVDU) for progressive and regressive motion, and an optomotor response detector. The optomotor response detector heavily inhibits the AVDUs in their non-preferred directions providing strong direction sensitivity. The motion detector array responds to increasing AV with a log-linear response as is found in electrophysiological recordings from the honeybee [21, 24]. This motion detector provides the input to the ring attractor both using the output of the AVDUs to drive changes based on the motion of the environment, but also by summation over smaller regions of the visual field to provide landmark responses.

### Ellipsoid body ring attractor network

Recent work has shown that the population activity of the EBw.s neurons in the ellipsoid body show dynamics and behaviour suggestive of a ring attractor network [11, 13]. A ring attractor is a form of linear attractor network where the connections loop across the two open ends to form a closed loop. Input due to the all-to-all inhibitory connectivity (*I*^*r*^) and input due to the local approximately Gaussian distributed excitatory connectivity ($E_{i}^{r}$) ensure a single bump of activity in the network. We chose a value for the Gaussian excitatory connectivity to match the bump full value half width (FVHW) measured by Seelig and Jayaraman [11]. The FVHW measured over 120s of simulation for our model are as follows (experimental values from [11] in brackets): single bar stimulus 82.7°±10.7° (82.3°±11.5°), panorama stimulus 85.8°±9.5° (84.9°±12.6°).

**Fig 2:**
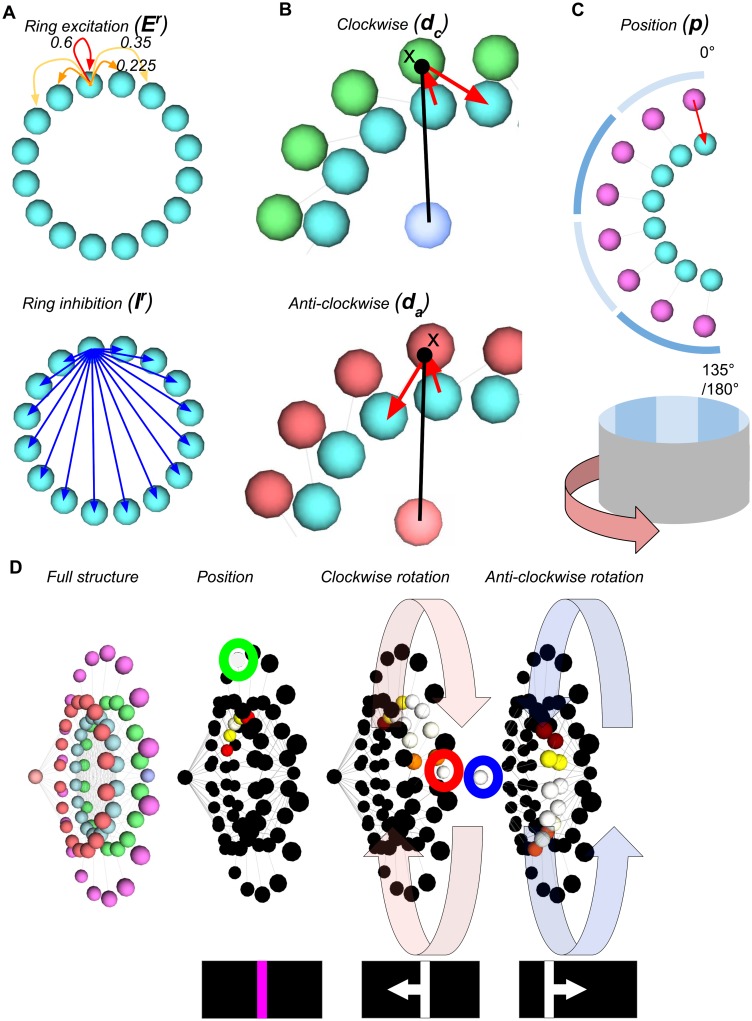
The ring attractor circuit. The components are as follows: Cyan: ring attractor neurons; Green: clockwise motion rotational neuron (with dark blue driver neuron); Red: anti-clockwise rotational neurons (with pale red driver neuron); Pink: Positional inputs. **A** shows the connectivity within the ring attractor for a single wedge, with local excitatory connections that decrease with distance, and uniform inhibitory connections. **B** shows the neural circuits for rotating the bump of activity around the ring clockwise and anti-clockwise for a single pair of wedges. The activity is gated by a single driver neuron (centre of the ring) which multiplies the ring activity to produce the output to the next wedge. **C** shows the positional input to one half of the ring for one neuron, and the angular mapping to the environment. **D** shows (left to right) the full structure of the ring; the ring being seeded by positional activity (green) to create a bump; the clockwise driver neuron (red) rotating the bump around the ring; the anti-clockwise driver neuron (blue) rotating the bump back to the initial location.

The ellipsoid body in *Drosophila* consists of a ring divided radially into 16 regions described as *wedges*, and we model each wedge as containing a single neuron of the ring attractor. This results in connectivity as shown in Fig 2.

The activity of this model is driven in two ways, as described in previous work using ring attractors to model vertebrate direction cells (e.g. [29–33]). The first is positional with an input corresponding to a region of the visual field for each wedge. The second is by the time derivative of position: the motion of the visual scene. For the former we only require input directly into the ring attractor however for the latter we must include additional neural circuitry. For the attractor to respond to motion we need two additional neuron types at each wedge location; one for clockwise rotations and one for anticlockwise rotations. These neurons take input from one wedge then output to the neighbouring wedge in the required motion direction. If these neurons have their activity gated multiplicatively by a global driver signal then the bump of activity on the ring will move in each direction in proportion to the strength of the driver signal for that direction. We describe the activity of neurons in the ring, clockwise rotation circuit, and anticlockwise rotation circuit as *r*_*i*_, *c*_*i*_ and *a*_*i*_ respectively for each wedge *i* up to *i*_*max*_. The positional inputs to the ring are *p*_*i*_ (See Eqs (3) and (4) for the static and plastic versions of this input) weighted by a global factor *w*_*p*_, and the clockwise and anticlockwise driver signals are *d*_*c*_ and *d*_*a*_. This gives the dynamics of the ring as:

$$\begin{array}{c} \frac{dr_{i}}{dt}=\frac{-r_{i}+E_{i}^{r}+I^{r}+w_{p}p_{i}+c_{i-1}+a_{i+1}}{\tau_{r}} \end{array}$$ (1)

$$\begin{array}{c} E_{i}^{r}=0.6r_{i}+0.35\left(r_{\left(i+1\right)\;\mathrm{mod}\;i_{max}}+r_{\left(i-1\right)\;\mathrm{mod}\;i_{max}}\right)+0.225\left(r_{\left(i+2\right)\;\mathrm{mod}\;i_{max}}+r_{\left(i-2\right)\;\mathrm{mod}\;i_{max}}\right) \end{array}$$ (2)

$$I^{r}=-0.1\sum_{0}^{i_{max}}r_{i}$$

$$\begin{array}{c} a_{i}=d_{a}r_{i} \end{array}$$

$$\begin{array}{c} c_{i}=d_{c}r_{i} \end{array}$$

The speed with which activity in the ring responds to the driver signals is determined by the time constant of the ring neurons, *τ*_*r*_, which is chosen as 1ms. The numbers in the equation for $E_{i}^{r}$ (Eq (2)) describe the weights of the approximate Gaussian distributed excitatory connections.

### Connections between motion detector and ring attractor

The ring attractor is driven by activity from the motion detector in two ways. First motion is summed across the entire field of view and forms clockwise and anti-clockwise drivers (*d*_*c*_ and *d*_*a*_) of the ring attractor. These drivers combine the AVDU outputs in the progressive and regressive directions across both eyes (*pg*_*R*_, *rg*_*R*_, *pg*_*L*_ and *rg*_*L*_) to form signals for leftward and rightward rotational motion. The driver signals are then multiplicatively (for mechanism see Nezis et al 2011 [34]) combined with the neighbouring ring attractor activity in a leaky integrator with time constant *τ*_*y*_ = 0.1*ms* such that:

$$\begin{array}{c} \frac{dd_{c}}{dt}=\frac{-d_{c}+pg_{R}+rg_{L}}{\tau_{y}} \end{array}$$

$$\begin{array}{c} \frac{dd_{a}}{dt}=\frac{-d_{a}+pg_{L}+rg_{R}}{\tau_{y}} \end{array}$$

Secondly, the ring attractor is driven by positional information. This information is derived from wide receptive fields evenly spaced across the horizontal field of view. These are designed to mimic the scale of receptive fields known to exist in *Drosophila* CX R4d ring neurons [14]. We use receptive fields, symmetric across the eyes, defined for each eye (*L*eft and *R*ight) by horizontal neuron ranges (with implicit summation down vertical columns) *x*_*k*_ and fixed weights, which divide the eye into a vertical set of stripes each with a three ommatidia width:

$$\begin{array}{c} RF_{j}^{\left\{L,R\right\}}=\sum_{k=3k}^{3k+2}x_{k}:j=0:7 \end{array}$$

These receptive fields are connected to the ring attractor retinotopically for the fixed weight experiments, as shown in Fig 2, with a global weighting factor *w*_*p*_ via positional cells. We fix the weights for the initial experiments to investigate the interactions of the motion and positional signals as well as the behaviour of the neural compass with each individually. These experiments provide context for the plastic weight experiments. They are governed by the following equation with time constant *τ*_*p*_ = 10*ms*:

$$\begin{array}{c} \frac{\text{d}p_{i}}{\text{d}t}=\{\begin{array}{ll} \left(-p_{i}+RF_{i}^{L}\right)/\tau_{p} & :i<i_{max}/2\\ \left(-p_{i}+RF_{i_{max}/2-i}^{R}\right)/\tau_{p} & :i\geq i_{max}/2 \end{array} \end{array}$$ (3)

Fixed RFs provide a suitable method for driving the compass however they do not agree with the experimental data of Seelig and Jayaraman [11]. Here we present a model hypothesising that RFs can be mapped onto compass positions in the EB ring attractor via plasticity. We then will test this model with the stimuli presented to the real *Drosophila*, where the accuracy of the neural compass differed depending on the nature of the stimuli, to see if our model presents the same deficits as the real flies do.

To match the proportions of inhibitory and excitatory neurotransmitters in the neurons projecting to the EB, where there are many neurons that are stained for the presence of GABA but fewer for excitatory neurotransmitters, we propose a ‘winner-takes-all’ structure to the input landmark fields. In this each landmark provides inhibitory connections to the other landmarks and excitatory plastic connections to all the EB.ws ring attractor neurons. The outputs of the excitatory landmark neurons *p*_*i*_ are therefore modelled as follows, where the action of the inhibitory neurons is subsumed as a summed inhibitory input applied in the EB ring and neuron activation is always positive.

$$\frac{\text{d}p_{l}}{\text{d}t}=\{\begin{array}{ll} \left(-p_{l}+RF_{l}^{L}-10\sum_{m\in \left[m\neq l\right]}p_{m}\right)/\tau_{p} & :l<l_{max}/2\\ \left(-p_{l}+RF_{l_{max}/2-l}^{R}-10\sum_{m\in \left[m\neq l\right]}p_{m}\right)/\tau_{p} & :l\geq l_{max}/2 \end{array}$$ (4)

A Hebbian learning rule with rapid presynaptic weight normalisation [35] is used to learn a mapping between the landmarks and the ring neurons. The learning rule is also driven by a ‘consolidation’ process where the difference between the sum of the presynaptic weights (which is normalised to a constant, *θ*) and the individual weight drives learning alongside the Hebbian learning by setting the threshold between long-term depression (LTD) and long-term potentiation (LTP) regimes. This consolidation is required as we seek to bind the landmarks to activity in the ring attractor that is not initially generated by the output of the landmark neurons, and standard Hebbian rules fail in these conditions [36, 37]. We will investigate two boundary conditions of this rule: one where the rate of the consolidation process is twice the rate of the Hebbian process and one where it is half.

These excitatory landmark neurons are then connected to the EB.ws neurons by plastic weights *w*_*li*_ which vary according to the rule:

$$\begin{array}{c} \theta =\sum_{m=0}^{m=i_{max}}w_{ml}=1.0 \end{array}$$

$$\frac{\text{d}w_{il}}{\text{d}t}=\{\begin{array}{ll} 0 & :w_{il}\leq 0\\ \alpha p_{l}\left(r_{i}-\beta \left(\theta -\gamma w_{il}\right)\right) & :w_{il}>0 \end{array}$$

where *i* is the postsynaptic index, *l* is the presynaptic index, *α* = 0.002 is the global learning rate, *β* = [0.5, 2] is the consolidation learning factor, *γ* = 1.1 is the stability of learned weights, and *β*(*θ* − *γw*_*il*_) sets the LTP/LTD threshold. These weights are then multiplied by the global landmark weight *w*^*p*^ = 0.02.

### Methods

#### Basic attractor responses (Experiments 1 & 2)

To test the basic response of the ring attractor we investigate the behaviour when driven by motion, position, and a combination of the two. This allows us to look at the accuracy of the individual systems, and the accuracy when they are combined. In addition, we can look at the accuracy that different numbers of positional RFs provide, which will provide context when investigating the accuracy of the model in Experiment 3, where plasticity is involved in the positional pathway.

For the initial experiments we use the *RF* receptive fields with *w*_*p*_ = 0.1 for position only, and *w*_*p*_ = 0.01 for position and motion, as in the absence of a motion signal a greater positional input is required to move the bump of activity in the ring attractor. The simulation timestep for all experiments is 0.1ms, and 120s are simulated.

Since our model will not generate its own turning movements for this set of experiments we drive the movement of the model using a temporally smoothed (time constant *τ*_*ϕ*_ = 100*ms*) summed Gaussian, random, process *N* ($\bar{x}=0$, *σ*^2^ = 10), where the change in absolute horizontal rotation (yaw, or azimuthal rotation), of the model, *ϕ* is given by:

$$\begin{array}{c} \frac{dN}{dt}=N\left(0,10\right) \end{array}$$

$$\begin{array}{c} \frac{d\phi }{dt}=\frac{-\phi +N}{\tau_{Az}} \end{array}$$

To compare the model ring attractor representation of azimuthal rotation with the simulated azimuthal rotation we take a vector average of the activity of the ring attractor. We weight the vector formed from the centre of the ring to each neuron’s location in the ring (**v**_*i*_) by that neuron’s activity (*r*_*i*_) and sum the vectors to give a resultant direction **v**_*sum*_. We then take the estimated azimuth angle, *ϕ*_*est*_, from this vector to the initial forward direction **v**_*init*_ and account for prior complete rotations by counting the transitions past 180° (*N*_*trans*_).

$$\begin{array}{c} v_{sum}=\sum_{i=0}^{i_{max}}r_{i}v_{i} \end{array}$$

$$\begin{array}{c} \phi_{est}=\arccos\left({\hat{v}}_{sum}\cdot {\hat{v}}_{init}\right)+2\pi N_{trans} \end{array}$$

We present a black environment with a single grey bar. The bar subtends 11.5° of visual angle in the horizontal direction, has infinite extent in the vertical direction, and has a luminance of 0.8 out of a maximum of 1.0. The model controls the azimuthal rotation of a virtual insect within this environment with fixed location. This mimics the environment presented to restrained behaving *Drosophila* in experiments [11]. The field of view of the model insect is described in the section ‘Motion detection’.

We process the data to compute the circular mean and standard deviation of the difference between the estimated and simulated azimuthal rotations using the Matlab Circular Statistics Toolbox [38]. As there is a processing delay in the ring attractor responding to changes in the azimuth of the simulated insect we calculate the standard deviation using offsets in the data from 0 to 60ms in 1ms increments, and use the offset with the minimum standard deviation.

#### Learned landmark attractor responses (Experiment 3)

This experiment investigates the way in which the motion and position pathways interact when the positional information must be learned. This motivation for this version of the model arises from the experimental evidence in Seelig and Jayaraman [11] that the activity in EB.w.s neurons has an angular offset to the position of the landmarks on the visual field which can change over long time periods for a single fly. This evidence indicates that the positional RFs do not map linearly onto the ring attractor but that the mapping may be plastic. We choose two parameterisations of the learning rule, and these affect the speed of the consolidation part of the learning rule (see Section *Landmark learning*) but not the Hebbian learning part of the rule. One parameterisation reduces the speed of the consolidation process and thus is closer to a pure Hebbian rule where the weights can change their mapping more easily. The other parameterisation increases the speed of consolidation which produces more stable weight mappings that require significant contradictory activity to change.

The behavioural protocol for Experiment 3 is the same as in Experiments 1 and 2 with a random Gaussian distribution driving the heading of the model within the environments.

**Fig 3:**
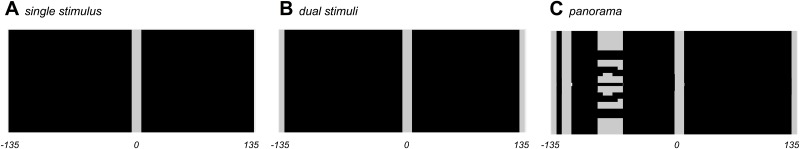
Stimuli for the model. **A** Single stimulus. **B** Dual stimuli. **C** Panorama of stimuli. These stimuli are based on the angular dimensions of the layouts used by Seelig and Jayaraman [11].

Three stimulus environments were used for this experiment: single bar, dual bar and panorama. These are shown in Fig 3. These environments are chosen to replicate the environments used by Seelig and Jayaraman [11] with only the dual bar experiment exhibiting rotational symmetry. The ‘models as animals’ protocol is used for this experiment, where identical naïve models are presented with one of the stimulus environments, and the difference in the random walk taken determines the learning that occurs. For Experiment 3 simulations are 60s long, with 10s given for learning to occur, and performance analysis being presented for the following 50s. The simulation timestep is 0.1ms.

#### Statistical analyses

Correlation: Correlation analyses were performed using Pearson’s test, as used in Seelig and Jayaraman’s analysis [11] in GNU Octave using the corr() command [39].

Non-parametric multi-sample test: non-parametric multi-sample test analyses were performed using the Matlab Circular Statistics Toolbox [38] function circ_cmtest(). For comparing between two simulation runs in Experiment 1 the difference between the actual azimuth and the ring attractor azimuth was calculated for each time point, and time points were sampled at 100ms intervals to provide independent samples for analysis. For analysis between stimuli in Experiment 3 the set of standard deviations of all simulation runs for a stimulus were taken as the independent variable sets.

Clustering: The distribution of the weights from the RFs to the ring attractor were analysed using clustering. The weights from each RF to the ring attractor neurons were taken as vectors with direction denoted by the target ring attractor neuron and magnitude denoted by the weight. These vectors for each RF were then averaged to give the resultant preferred direction for that RF. The direction of each RF was then subtracted from the direction that RF would map to if the RFs were arranged in an even retinotopic mapping: thus RF directions corresponding to a single retinotopic mapping would now have similar directions after subtraction. All RF vectors were then next set to unit length, and decomposed into cosine and sine components. These components were then clustered using 2D clustering via the DBSCAN algorithm implementation from Scikit-learn [40] with *ϵ* = 0.25.

## Results

### Experiment 1: The activity in the ring attractor tracks rotation of a single moving bar with greater accuracy if position and motion are combined

We ran the model for 120s (at 0.1ms timesteps) of simulation time for three conditions: motion input to the ring attractor only; position input using *RF* receptive fields to the ring attractor only; combined motion and position input, with the position input reduced tenfold so it does not dominate.

**Fig 4:**
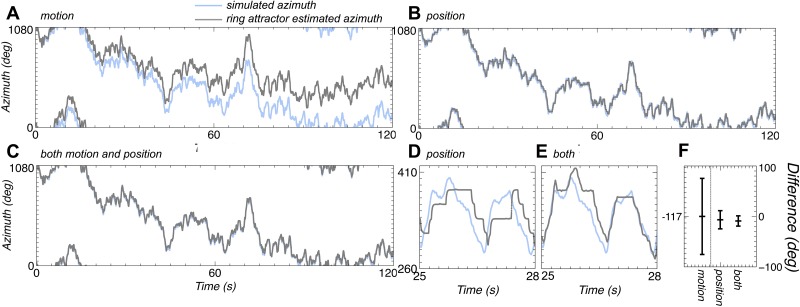
Ring attractor azimuth estimate accuracy. **A** shows the simulated (blue) and estimated (grey) azimuthal rotations when the ring attractor is driven only by the motion of the bar. There is overall good tracking for much of the simulation however there are time periods where the estimate drifts away from the simulated value. **B** show the same rotations when positional cues drive the ring attractor. There is better tracking, however **D** shows that the width of the receptive fields leads to a stepped profile for the ring attractor estimate as movement of the bar within a receptive field cannot be detected. **C** shows the same rotations with combined position and motion driving the ring attractor. There is good tracking, and **E** shows that the motion signal can compensate for the insensitivity of the motion system within a receptive field. **F** summarises the results showing that the circular mean and standard deviation (calculated using the Matlab Circular Statistics Toolbox [38]) of the difference between the simulated and estimated rotations is largest with motion only driving the ring attractor and smallest with combined motion and position.

Fig 4 shows the azimuthal rotation estimated by the model ring attractor and the actual azimuthal rotation of the virtual insect, for each of these conditions. In addition the mean and standard error of the difference between the two azimuth values is shown. The Pearson’s correlation between the two azimuthal rotation measures is high (*R* > 0.97) in all conditions. There is a clear improvement in the correspondence between the two azimuth values when position cues are used, and even greater improvement when position and motion are combined. It should be noted, however, that the improvement of motion and position over position alone is small. A non-parametric multi-sample test (see Methods for details) shows that all differences in the distributions of the difference between the ring attractor azimuth and actual azimuth between conditions are statistically significant (all *p* ≪ 0.01 except position: motion and position (*p* < 0.05)).

### Experiment 2: More positional receptive fields provide more accurate tracking of the bar location

**Fig 5:**
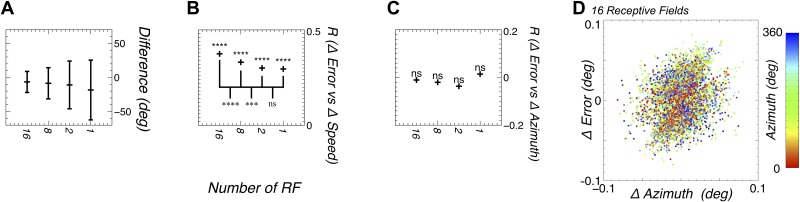
Receptive field number correlates with tracking accuracy in the ring attractor. **A** Greater numbers of receptive fields provide a reduction in the circular standard deviation (calculated using the Matlab Circular Statistics Toolbox [38]) of the error between the actual azimuth and the estimate from the ring attractor. **B** The correlation between the change in the tracking error and the change in azimuth shows significant positive correlation, which is significantly different for larger RF numbers than for smaller RF numbers. **C** The correlation between the change in tracking error and the absolute azimuth shows no significant correlation (see main text for details of the test). **D** An example of the correlation between the change in tracking error and the change in azimuth (for 16 RF, data sampled every 1000 iterations, using the temporal offset calculated to find the mean azimuthal offset). The colour of each point indicates the absolute azimuth, showing no correlation to error change. One example is used as it is representative of all such plots.

To test the influence that the number of receptive fields have on the performance of the tracking accuracy of the ring attractor we ran the model with differing numbers of receptive fields. This was performed by removing an evenly spaced proportion of the receptive fields, by halving the number of fields, by providing one eighth the number of fields, or in the final case by only providing one receptive field. The paradigm otherwise remained identical to that in Experiment 1. Fig 5 shows the results of these experiments. It can be seen that there is a decrease in accuracy as the number of receptive fields decreases however even one receptive field provides much greater accuracy than when no receptive fields (i.e. motion input only) are used. A non-parametric multi-sample test (see Methods for details) shows that all differences are highly statistically significant due to the size of the data set (*p* ≪ 0.01). The Pearson’s correlation between the two azimuthal rotation measures is high (*R* > 0.99) in all conditions.

### Experiment 3: A Hebbian rule allows learning of the mapping of landmarks to the ring attractor

We next tested the ability of our Hebbian based learning rule to associate the position of landmarks in the visual world with positions on the neural compass with two parameter variants, chosen by a limited exploration of the parameter space for the rate of weight consolidation, *β*. The two parameterisations show two distinct modes of learning for the model, one that creates a retinotopic mapping that can change once established (*β* = 0.5) and one which creates a fixed retinotopic mapping (*β* = 2.0). These values are chosen as examples as more extreme values of *β* fail to create retinotopic mappings and match the experimental data. The stimuli used to test the model were replications of those used by Seelig and Jayaraman [11], consisting of a single bright vertical bar on a dark background, two bars separated by 135° horizontally (thus displaying 2-fold rotational symmetry in the 270° visual world), and a panorama of four bars with no rotational symmetry, as shown in Fig 3.

**Fig 6:**
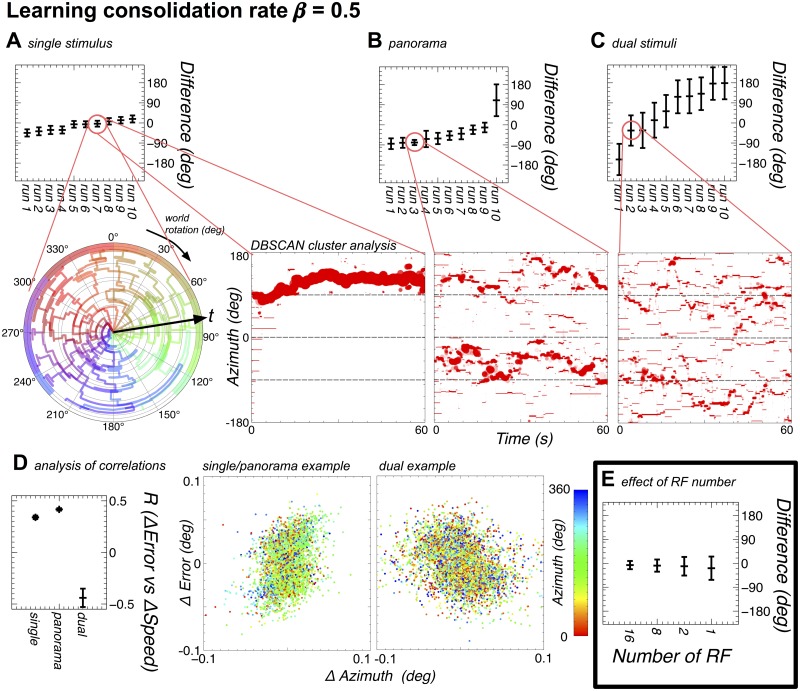
The model can learn to map landmarks to positions on the ring attractor with slow consolidation (*β* = 0.5) of the plastic weights. In addition the evolved offsets when the ring attractor is seeded to one position are shown. **A** The model performance (see **E** for comparison with the results from Experiment 2) in terms of mean and circular standard deviation (calculated using the Matlab Circular Statistics Toolbox [38]) of the ring attractor direction from the world direction (corresponding to the location of the single stimulus, and remapped from 270° to 360°) is shown at the top, and shows that the model is able to track the motion of the world well in all cases, with an offset to the position of the stimulus on the visual field. Additionally, a polar plot contains an example of the evolution of the weights over the course of the simulation (with time increasing from the centre to the outside) for a single stimulus. In the polar plot each receptive field (RF) is given a colour, with the key around the outside of the ring, and the angular position of each line denotes the position on the ring attractor that each RF maps to. An ordered mapping, with no offset, should therefore be shown by each line lying in the circular segment under the corresponding key item. Instead, we see that the weights remap the 260° world onto approximately 360° of the ring attractor, and the learning is established early in the simulation, although the weights do show changes in the mapping over the course of the simulation. A second Cartesian plot shows the clustering of the RFs into retinotopic maps (see Methods for details), with the size of the marker denoting the number of RFs in a cluster. For the single stimulus it can be seen that a single map evolves, but changes offset over time. **B** As A, but with the panorama. The polar plots are not helpful for the panorama as there are multiple retinotopic mappings developed. Here there is once again a good performance in tracking the world in most cases, albeit with one run where tracking performance is poor. The example showing the weight clustering shows that multiple retinotopic mappings are developed, however these mappings change over the course of the simulation. **C** As A, but with dual stimuli. In this case the model exhibits poor performance tracking the world, and the weights remain largely changeable throughout the simulation. **D** This panel shows the correlations between the changes in azimuthal position and the changes in the offset between the actual azimuth and the azimuth represented on the ring attractor. The single and panorama simulations show a similar correlation to that found with fixed RFs, however the dual stimuli simulations show a negative correlation between the change in position and the change in error.

**Fig 7:**
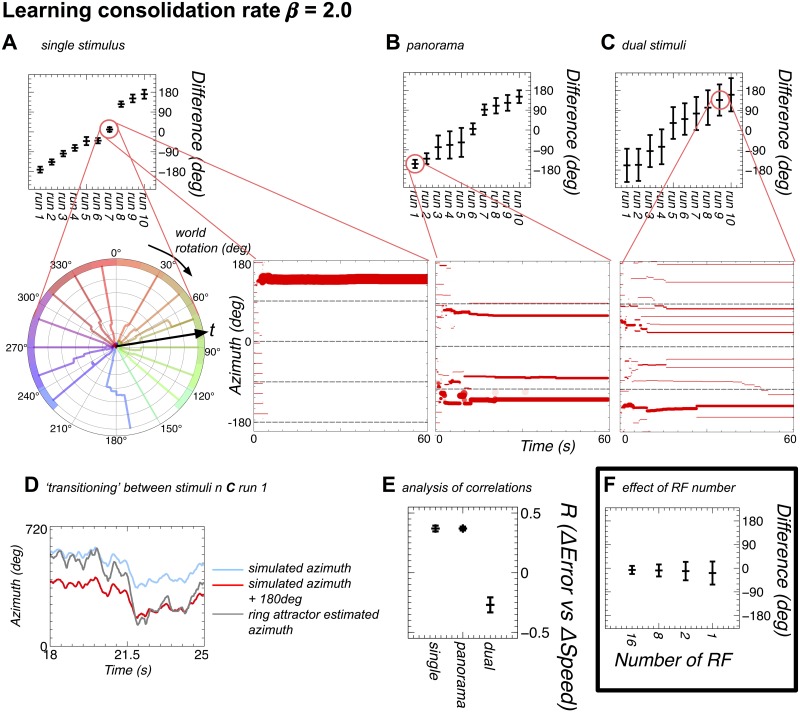
The model can learn to map landmarks to positions on the ring attractor with fast consolidation (*β* = 2.0) of the plastic weights. In addition the evolved offsets when the ring attractor is seeded randomly are shown. **A** As Fig 6: **A** except with *β* = 2.0. Here the weights finish evolving early on in the simulation and remain fixed after that point. There is only one retinotopic mapping developed. **B** As Fig 6: **B** except with *β* = 2.0. The weights form three retinotopic mappings, which persist for the rest of the simulation. **C** As Fig 6: **C** except with *β* = 2.0. The weights form many weak mappings, with few RFs belonging to any one mapping. **D** This panel shows ‘transitioning’ between stimuli as found in the experimental data of Seelig and Jayaraman [11]. **E** This panel shows the correlations between the changes in azimuthal position and the changes in the offset between the actual azimuth and the azimuth represented on the ring attractor. The single and panorama simulations show a similar correlation to that found with fixed RFs, however the dual stimuli simulations show a negative correlation between the change in position and the change in error.

Figs 6 & 7 shows the results for all these three cases for each parameterisation, with examples showing the time evolution of the landmark weights, and the circular mean and standard deviation for the final 50s of the 60s simulation. Interestingly, despite the marked differences in the evolution of the weights, a non-parametric multi-sample test (see methods for details) shows low or no significance in the difference (*p* > 0.07, except dual bar (*p* ≫ 0.1)) between the distributions of standard deviations for the two values of *β*. There are, however, significant differences between the distributions of standard deviations for the different stimuli in both cases (all *p* ≪ 0.01). The Pearson’s correlation between the two azimuthal rotation measures is high (*R* > 0.99) in all conditions. In keeping with the results from Experiment 2, Pearson’s correlation analysis of change in tracking error and the absolute azimuthal position showed no significant correlation between these data for any simulation run.

#### Parameter variant 1: *β* = 0.5

With a purer Hebbian form of learning we see that only in the case of the single bar stimulus set does the model maintain weight mapping, and there is variation even in this mapping over time. With the panorama stimuli the model changes between mappings throughout the trial (evidenced by the opposing green and purple at fixed radial distances). The model does maintain a fixed offset between the ring attractor direction and the world rotation in almost all cases, but this is driven by the changing weight mappings. In the case of the dual stimuli the model forms a rough and extremely variable mapping, and the neural compass ‘transitioning’ between the stimuli degrades the performance.

#### Parameter variant 2: *β* = 2.0

This variant of the learning rule forms more stable weight mappings, and this is clear from the polar weight plots. In all cases for the single bar and panorama stimuli sets it is clear that the model is able to learn stable weight mappings, albeit with a fixed offset between the neural compass and the visual stimuli. There is a notable difference between the mapping learned in the single stimulus case and that learned from the panorama stimuli. The single stimulus rapidly produces a clear mapping, which does not alter after the first few seconds. The panorama stimuli show less specificity, with some RFs mapping to different offsets of the panorama relative to the model head direction.

In the case of the two bar stimuli, there is weaker tracking, and an analysis of the individual trials shows that this is in part due to the neural compass ‘transitioning’ between tracking each of the two bars in the environment. This is consistent with the behaviour found by Seelig and Jayaraman [11]. The cluster weight plot shows a very non-topographic global mapping, due to many different mappings being laid over each other. These mappings are, however, fixed for most of the simulation and therefore the source of the ‘transitioning’ is not due to changes in weights. The angular distance between the mappings indicates that one source of the transitions could be the bump activity transitioning between being driven by each of two different mappings, however it should also be noted that transitioning can also be caused by a given mapping moving from tracking one stimulus to tracking the other stimulus. Analysis of the distribution of the offset between the actual azimuth and ring attractor azimuth does not resolve this ambiguity.

## Discussion

The central complex in insects is an intriguing structure, which is only beginning to be understood. Centrally positioned, ancient [41] and the source of massive sensory convergence, it is well placed to perform a range of tasks. It is therefore not surprising that it has been implicated as having a role in locomotion [4], courtship [2], visual pattern memory [6] and polarisation detection [8]. We present a model here that frames what we do know about the central complex, that it is the site of activity that tracks the heading of the insect, and we investigate the computational mechanisms behind this activity and provide mechanistic explanations for features of the experimental data that were previously not understood. While this study does not provide a conclusive summary of the role of the central complex, it provides the basis for more elaborate models.

Previous modelling work investigating head position cells in vertebrates, notably the rat, has mainly focused on the self organisation of ring attractor networks based on associative learning rules (e.g. [30–33]), which is not a concern in the insect as there is a well established morphology that contains the connections required for a ring attractor [42]. Learning of landmarks is mentioned, but not computationally evaluated [29]. We therefore present a model that does not focus on the dynamics of the ring attractor, but instead on the signals that drive it and their association with landmarks in the visual world.

It is notable that the performance of the model in the single stimulus trials is equivalent to the performance of the fixed RF model with 16 RFs for both learning rule parameterisations. This shows that fixed RFs provide less flexibility for equivalent performance.

Another key point is that there is still a spread of offsets between the neural compass and the real world landmarks found in the model behaviour when the ring attractor is seeded with activity at a fixed position: demonstrating that there is no implicit fixed bias to the offset induced by the starting state of the ring attractor.

The two parameterisations of the learning rule show similar behaviour of the neural compass but very different outcomes in the evolution of the plastic weights over the simulation. This shows that there are a range of parameter values for *β*, the consolidation speed, that can reproduce the experimental findings. To separate the likelihood of these two values of *β* being closer to mechanisms in the real fly we can look to the possible role of the ring attractor as a neural compass. If a compass is to be useful for long-term navigation as found in place learning [7] then the weights must be resistant to change, so for such a role we favour the hypothesis that the real mechanism will be closer to the model with *β* = 2.0.

Finally, and most importantly, the role of the motion signal becomes clear when landmark learning is considered. Previously we have seen that the motion signal provides a poor substitute for positional information, accumulating errors rapidly. In addition the motion signal provides little increase in accuracy over a purely positional system. The model therefore predicts that these are not the reasons for a motion signal in the CX. The model instead predicts that the motion signal provides a required training signal when the landmarks are being learned, allowing neighbouring landmarks to trigger neighbouring neurons in the neural compass, as the activity in the ring attractor is driven initially by motion only. This explains the existence of the two systems, as motion cannot represent direction without drift, but absolute direction cannot be learned without the motion signal.

In summary, our model makes the following predictions of several features found in the experimental data.

1. Few receptive fields (RF) are required for accurate tracking of rotation, and the number of RFs required for good rotation tracking aligns with the numbers of visual neurons converging on the central complex in the fly [14, 43].
2. As found in the data, dual stimuli will result in poor performance, and that ‘transitioning’ will occur between tracking each of the stimuli.
3. There will be a range of offsets between the location of the stimuli on the visual field and the neural compass, and that the 270° arena is mapped onto the full ring attractor.
4. Finally, our model predicts that rotational, non-positional information is present in the central complex because it is an essential part of the process of learning how to associate RFs with compass directions. It is not to provide a backup form of rotation tracking (as it accumulates errors rapidly), or to improve tracking with a learned positional mapping (as the improvement it provides is small).

We acknowledge that the current study has limitations. One is that, in the absence of motion activity, the activity in the ring attractor is drawn to stable states centred on the neurons in the ring attractor circle. Since there are only 16 neurons in our attractor, this means that inaccuracies can accrue rapidly. When driven with a random Gaussian process these inaccuracies average out over the course of the simulation, however when driven by more purposeful behaviour they can significantly affect the behaviour of our model. It is possible that a more complex ring attractor could be devised that does not suffer from these stable states. Additionally some aspects of model behaviour are difficult to compare to the experimental data. For example, Seelig and Jayaraman compute the correlation between the angle indicated in the central complex and the heading of the fly, and find that there is a variability in correlation between runs. In our model the correlation is always extremely high (*R* > 0.99), and it is uncertain whether this difference is due to noise introduced by the recording mechanism used by Seelig and Jayaraman, or differences between the model and the biological system. A similar argument also applies where we do not find more than one activity bump on the ring attractor at any point, while Seelig and Jayaraman occasionally do.

Does the central complex contain a compass or not? The model indicates that it is necessary with the simple learning shown here to bind direction to the angular position of a single significant feature in the environment. This means that as the *in silico* animal moves around, the location of this feature on the ring attractor can move if it is not far away. This means that the ‘compass’ is not a compass in the sense we mean but more akin to a magnetic compass near the poles: it points to a single significant feature in the current environment. This is a change in the role of the ring attractor, but for simple environmental associative learning does not remove the usefulness of the system. For example, so long as the ring attractor is consistent in direction for a given environment, and given an additional means of determining location based on multiple sensory features, a mechanism driving behaviour towards a rewarded heading will still be useful. Such a mechanism allows that the ‘compass’ is local but requires a positional system (or place memory) to work in combination with.

The absence of a global compass does constrain certain behaviours found in insects, notably long range scouting and navigation, which require a form of path integration. It is therefore interesting to note that honeybees only forage at previously visited locations close to home under cloudy skies [44] when absolute direction information from UV light polarisation is not available to them. Such behaviour does suggest that a local compass, as found here, may be sufficient for place-based navigation as is found in *Drosophila* and provides an avenue for better understanding such behaviours.

The existence of the compass, and the understanding of how it is implemented, provided a first step in understanding the central complex, and this work adds to complementary studies that unearth the possible computational mechanisms at play in the central complex (path integration [3, 45], the celestial compass [9], and place finding [7] for example).

The possibilities unleashed by understanding the CX far exceed an understanding of brain function in insects. Such understanding may also ultimately lead to the development of algorithms for advanced robotic autonomy: giving robots the ability to rapidly orient themselves within a new environment and associate headings relative to landmarks with behavioural outcomes. This ability would permit goal directed behaviour with greater autonomy than is currently possible and, given the abilities of more advanced insects (notably *Hymenoptera*) to navigate over long distances, would provide the basis for the development of more advanced navigation.

## References

[1] Utting M, Agricola H, Sandeman R, Sandeman D. Central complex in the brain of crayfish and its possible homology with that of insects. J Comp Neurol. 2000;416(2):245–61. 10.1002/(SICI)1096-9861(20000110)416:2<245::AID-CNE9>3.3.CO;2-1 10581469

[2] Homberg U. Evolution of the central complex in the arthropod brain with respect to the visual system. Arthropod Struct Dev. 2008;37(5):347–62. 10.1016/j.asd.2008.01.008 18502176

[3] Wessnitzer J, Webb B. Multimodal sensory integration in insects—towards insect brain control architectures. Bioinspir Biomim. 2006;1(3):63–75. 17671308 10.1088/1748-3182/1/3/001

[4] Strauss R. The central complex and the genetic dissection of locomotor behaviour. Curr Opin Neurobiol. 2002;12(6):633–638. 10.1016/S0959-4388(02)00385-9 12490252

[5] Kathman ND, Kesavan M, Ritzmann RE. Encoding wide-field motion and direction in the central complex of the cockroach Blaberus discoidalis. J Exp Biol. 2014;217(22). 10.1242/jeb.112391 25278467

[6] Wang Z, Pan Y, Li W, Jiang H, Chatzimanolis L, Chang J, et al Visual pattern memory requires foraging function in the central complex of Drosophila. Learn Mem. 2008;15(3):133–42. 10.1101/lm.873008 18310460 PMC2275655

[7] Ofstad TA, Zuker CS, Reiser MB. Visual place learning in Drosophila melanogaster. Nature. 2011;474(7350):204–7. 10.1038/nature10131 21654803 PMC3169673

[8] Labhart T, Meyer EP. Neural mechanisms in insect navigation: polarization compass and odometer. Curr Opin Neurobiol. 2002;12(6):707–714. 10.1016/S0959-4388(02)00384-7 12490263

[9] Homberg U, Heinze S, Pfeiffer K, Kinoshita M, el Jundi B. Central neural coding of sky polarization in insects. Philos Trans R Soc London B Biol Sci. 2011;366 (1565). 10.1098/rstb.2010.0199 21282171 PMC3049008

[10] Taube J, Muller R, Ranck J JB. Head-direction cells recorded from the postsubiculum in freely moving rats. I. Description and quantitative analysis. J Neurosci. 1990;10(2):420–435. 2303851 10.1523/JNEUROSCI.10-02-00420.1990 PMC6570151

[11] Seelig JD, Jayaraman V. Neural dynamics for landmark orientation and angular path integration. Nature. 2015;521(7551):186–191. 10.1038/nature14446 25971509 PMC4704792

[12] Varga AG, Ritzmann RE, Taube JS, Muller RU, Ranck JB, Taube JS, et al Cellular Basis of Head Direction and Contextual Cues in the Insect Brain. Curr Biol. 2016;26(14):1816–28. 10.1016/j.cub.2016.05.037 27397888

[13] Turner-Evans DB, Jayaraman V, elÂ Jundi B, Warrant EJ, Byrne MJ, Khaldy L, et al The insect central complex. Curr Biol. 2016;26(11):R453–7. 10.1016/j.cub.2016.04.006 27269718

[14] Seelig JD, Jayaraman V. Feature detection and orientation tuning in the Drosophila central complex. Nature. 2013;503(7475):262–6. 10.1038/nature12601 24107996 PMC3830704

[15] Wolf R, Heisenberg M. Visual control of straight flight in Drosophila melanogaster. J Comp Physiol A. 1990;167(2):269–83. 10.1007/BF00188119 2120434

[16] Dill M, Wolf R, Heisenberg M. Behavioral analysis of Drosophila landmark learning in the flight simulator. Learn Mem. 1995;2(3–4):152–60. 10.1101/lm.2.3-4.152 10467572

[17] Wolf R, Heisenberg M. Visual space from visual motion: turn integration in tethered flying Drosophila. Learn Mem. 1997;4(4):318–27. 10.1101/lm.4.4.318 10706369

[18] Guo C, Du Y, Yuan D, Li M, Gong H, Gong Z, et al A conditioned visual orientation requires the ellipsoid body in Drosophila. Learn Mem. 2014;22(1):56–63. 10.1101/lm.036863.114 25512578 PMC4274327

[19] McCann GD, MacGinitie GF. Optomotor Response Studies of Insect Vision. Proc R Soc B Biol Sci. 1965;163(992):369–401. 10.1098/rspb.1965.0074 4378844

[20] Kirchner WH, Srinivasan MV. Freely flying honeybees use image motion to estimate object distance. Naturwissenschaften. 1989;76(6):281–282. 10.1007/BF00368643

[21] Ibbotson MR. Evidence for velocity-tuned motion-sensitive descending neurons in the honeybee. Proc R Soc L [Biol]. 2001;268(1482):2195–201. 10.1098/rspb.2001.1770 PMC1088866 11674866

[22] Barron A, Srinivasan MV. Visual regulation of ground speed and headwind compensation in freely flying honey bees (Apis mellifera L.). J Exp Biol. 2006;209(Pt 5):978–84. 10.1242/jeb.02085 16481586

[23] Fry SN, Rohrseitz N, Straw AD, Dickinson MH. Visual control of flight speed in Drosophila melanogaster. J Exp Biol. 2009;212(Pt 8):1120–30. 10.1242/jeb.020768 19329746

[24] Cope A, Sabo C, Gurney KN, Vasislaki E, Marshall JAR. A Model for an Angular Velocity-Tuned Motion Detector Accounting for Deviations in the Corridor-Centering Response of the Bee. PLoS Comput Biol. 2016;. 10.1371/journal.pcbi.1004887 27148968 PMC4858260

[25] Pfeiffer K, Homberg U. Organization and functional roles of the central complex in the insect brain. Annu Rev Entomol. 2014;59:165–84. 10.1146/annurev-ento-011613-162031 24160424

[26] Richmond P, Cope A, Gurney K, Allerton DJ. From model specification to simulation of biologically constrained networks of spiking neurons. Neuroinformatics. 2013;12(2):307–23. 10.1007/s12021-013-9208-z PMC4003408 24253973

[27] Cope AJ, Richmond P, James SS, Gurney K, Allerton DJ. SpineCreator: A graphical user interface for the creation of layered neural models. In-press. 2015; 10.1007/s12021-016-9311-z PMC5306153 27628934

[28] Power ME. The effect of reduction in numbers of ommatidia upon the brain of Drosophila melanogaster. J Exp Zool. 1943;94(1):33–71. 10.1002/jez.1400940103

[29] Skaggs WE, Knierim JJ, Kudrimoti HS, McNaughton BL. A Model of the Neural Basis of the Rat\textquotesingle s Sense of Direction In: Tesauro G, Touretzky DS, Leen TK, editors. Adv. Neural Inf. Process. Syst. 7 MIT Press; 1995 p. 173–180. Available from: http://papers.nips.cc/paper/890-a-model-of-the-neural-basis-of-the-rats-sense-of-direction.pdf. 11539168

[30] Xie X, Hahnloser RHR, Seung HS. Double-ring network model of the head-direction system. Phys Rev E. 2002;66(4):041902 10.1103/PhysRevE.66.041902 12443230

[31] Stringer SM, Trappenberg TP, Rolls ET. Self-organizing continuous attractor networks and path integration: one-dimensional models of head direction cells. Comput Neural Syst. 2002;13:217–242. 12061421

[32] Stringer SM, Rolls ET, Trappenberg TP. Self-organizing continuous attractor network models of hippocampal spatial view cells. Neurobiol Learn Mem. 2005;83(1):79–92. 10.1016/j.nlm.2004.08.003 15607692

[33] Knierim JJ, Zhang K. Attractor Dynamics of Spatially Correlated Neural Activity in the Limbic System HD: head direction. Annu Rev Neurosci. 2012;35:267–85. 10.1146/annurev-neuro-062111-150351 22462545 PMC5613981

[34] Nezis P, van Rossum MCW. Accurate multiplication with noisy spiking neurons. J Neural Eng. 2011;8(3):034005 21572218 10.1088/1741-2560/8/3/034005

[35] Zenke F, Gerstner W. Cooperation across timescales between and Hebbian and homeostatic plasticity. Philos Trans R Soc London, Ser B Biol Sci. 2016; (preprint).

[36] Vasilaki E, Frémaux N, Urbanczik R, Senn W, Gerstner W. Spike-Based Reinforcement Learning in Continuous State and Action Space: When Policy Gradient Methods Fail. PLoS Comput Biol. 2009;5(12):e1000586 10.1371/journal.pcbi.1000586 19997492 PMC2778872

[37] Richmond P, Buesing L, Giugliano M, Vasilaki E. Democratic Population Decisions Result in Robust Policy-Gradient Learning: A Parametric Study with GPU Simulations. PLoS One. 2011;6(5):e18539 10.1371/journal.pone.0018539 21572529 PMC3087717

[38] Berens P. CircStat: A MATLAB Toolbox for Circular Statistics; 2009. Available from: http://www.jstatsoft.org/v31/i10/.

[39] John W Eaton David Bateman SH, Wehbring R. {GNU Octave} version 4.0.0 manual: a high-level interactive language for numerical computations; 2015. Available from: http://www.gnu.org/software/octave/doc/interpreter.

[40] Pedregosa F, Varoquaux G, Gramfort A, Michel V, Thirion B, Grisel O, et al Scikit-learn: Machine Learning in {P}ython. J Mach Learn Res. 2011;12:2825–2830.

[41] Strausfeld NJ, Hirth F. Deep Homology of Arthropod Central Complex and Vertebrate Basal Ganglia. Science (80-). 2013;340 (6129). 10.1126/science.1231828 23580521

[42] Wolff T, Iyer NA, Rubin GM. Neuroarchitecture and neuroanatomy of the *Drosophila* central complex: A GAL4-based dissection of protocerebral bridge neurons and circuits. J Comp Neurol. 2015;523(7):997–1037. 10.1002/cne.23705 25380328 PMC4407839

[43] Dewar ADM, Wystrach A, Graham P, Philippides A. Navigation-specific neural coding in the visual system of Drosophila. Biosystems. 2015;136:120–7. 10.1016/j.biosystems.2015.07.008 26310914

[44] Dyer FC, Gould JL. Honey bee orientation: a backup system for cloudy days. Science. 1981;214(4524):1041–2. 10.1126/science.214.4524.1041 17808669

[45] Haferlach T, Wessnitzer J, Mangan M, Webb B. Evolving a Neural Model of Insect Path Integration. Adapt Behav. 2007;15(3):273–287. 10.1177/1059712307082080

